# What Role Do Annelid Neoblasts Play? A Comparison of the Regeneration Patterns in a Neoblast-Bearing and a Neoblast-Lacking Enchytraeid Oligochaete

**DOI:** 10.1371/journal.pone.0037319

**Published:** 2012-05-16

**Authors:** Maroko Myohara

**Affiliations:** Insect Growth Regulation Research Unit, National Institute of Agrobiological Sciences, Tsukuba, Ibaraki, Japan; University of Sheffield, United Kingdom

## Abstract

The term ‘neoblast’ was originally coined for a particular type of cell that had been observed during annelid regeneration, but is now used to describe the pluripotent/totipotent stem cells that are indispensable for planarian regeneration. Despite having the same name, however, planarian and annelid neoblasts are morphologically and functionally distinct, and many annelid species that lack neoblasts can nonetheless substantially regenerate. To further elucidate the functions of the annelid neoblasts, a comparison was made between the regeneration patterns of two enchytraeid oligochaetes, *Enchytraeus japonensis* and *Enchytraeus buchholzi*, which possess and lack neoblasts, respectively. In *E. japonensis*, which can reproduce asexually by fragmentation and subsequent regeneration, neoblasts are present in all segments except for the eight anterior-most segments including the seven head-specific segments, and all body fragments containing neoblasts can regenerate a complete head and a complete tail, irrespective of the region of the body from which they were originally derived. In *E. japonensis*, therefore, no antero-posterior gradient of regeneration ability exists in the trunk region. However, when amputation was carried out within the head region, where neoblasts are absent, the number of regenerated segments was found to be dependent on the level of amputation along the body axis. In *E. buchholzi*, which reproduces only sexually and lacks neoblasts in all segments, complete heads were never regenerated and incomplete (hypomeric) heads could be regenerated only from the anterior region of the body. Such an antero-posterior gradient of regeneration ability was observed for both the anterior and posterior regeneration in the whole body of *E. buchholzi.* These results indicate that the presence of neoblasts correlates with the absence of an antero-posterior gradient of regeneration ability along the body axis, and suggest that the annelid neoblasts are more essential for efficient asexual reproduction than for the regeneration of missing body parts.

## Introduction

Recent advances in the field of stem cell biology are raising expectations that human regenerative medicine will become a future reality and are accelerating regeneration research in a range of model systems that utilize different regenerative strategies [1]. Among these model systems, planarians are bilaterian organisms that have the most prominent regenerative capabilities known and can reproduce a complete individual from only a small body fragment. This remarkable ability is due to the presence of adult somatic stem cells known as neoblasts [2]. Some types of annelids also exhibit regenerative abilities that are comparable to planarians, but this is thought to occur primarily through cellular dedifferentiation and redifferentiation, without the contribution of totipotent stem cells [3]. Hence, the elucidation of the regeneration mechanisms of annelids is expected to provide valuable information that will advance the exploration of the regenerative capabilities of vertebrates, as these also occur without the contribution of totipotent stem cells, and assist with developing the means to enhance these processes. Previously, we proposed the recently described fragmenting pot worm *Enchytraeus japonensis* as a new model system for regeneration studies [4]. *E. japonensis* reproduces asexually by dividing its body into several fragments, which then regenerate a complete individual within 4–5 days. Artificially amputated fragments of this organism that are as short as a few segments can also regenerate new individuals in the same manner.

The term ‘neoblast’ was first used more than a century ago to denote specialized cells that participate in the regeneration of mesodermal tissues in the oligochaete annelid *Lumbriculus* [5]. However, this nomenclature is now more often used to designate the pluripotent/totipotent adult somatic stem cells that play central roles in planarian regeneration [2], [6]–[8]. Recent advances in our knowledge of annelid regeneration from studies using *E. japonensis* as the model system [4], [9]–[14] have renewed interest in the long-ignored annelid neoblasts [15]–[17]. It must be noted however that planarian and annelid neoblasts are morphologically and functionally distinct.

**Figure 1:**
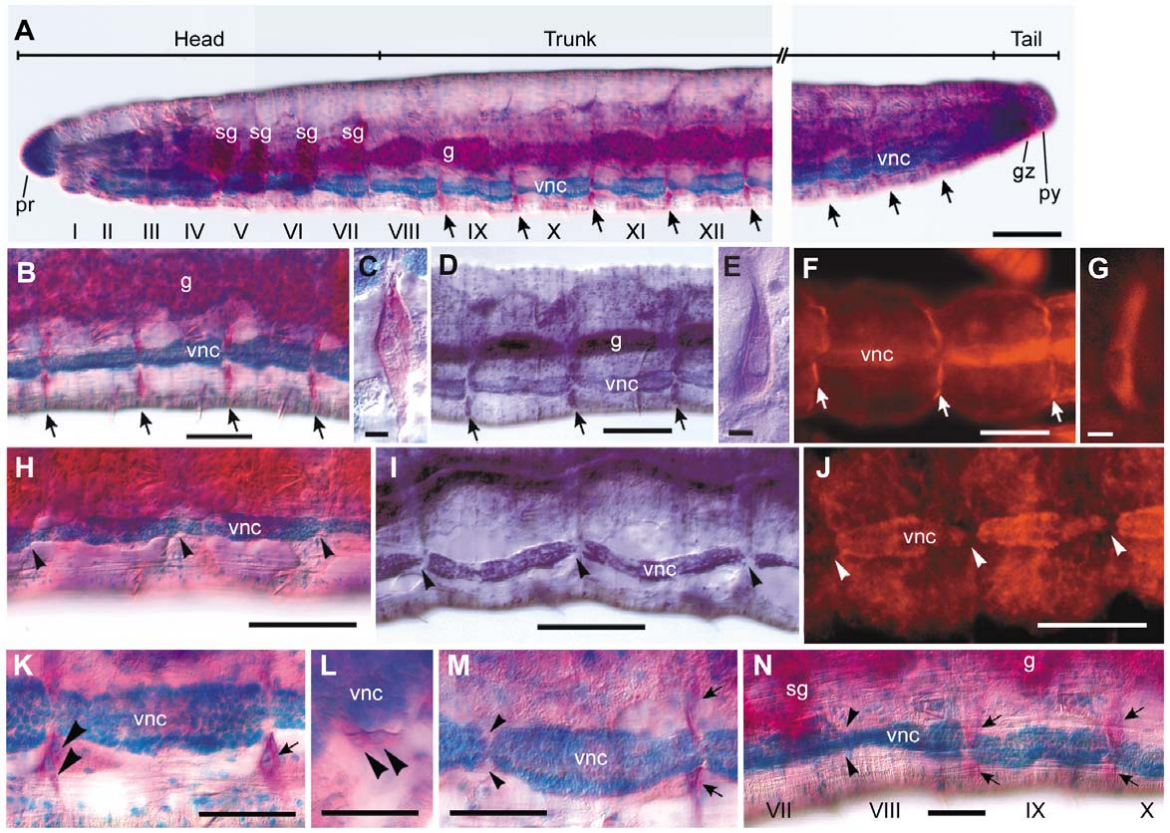
Distribution of neoblasts in *E. japonensis* and their absence in *E. buchholzi*. (A–G) Intact *E. japonensis* worms were stained with methyl green-pyronin (A–C), thionine (D–E), or propidium Iodide (F–G). Neoblast pairs (arrows) are localized on the intersegmental septa along the ventral nerve cord in all segments except for the eight anterior-most segments, i.e. the seven head-specific segments (segment I–VII) and the first trunk segment (segment VIII). (H–J) Neoblasts are absent from the corresponding position (arrowheads) in *E. buchholzi* as revealed by staining with methyl green-pyronin (H), thionine (I), or propidium Iodide (J). (K–N) Behavior of neoblasts (arrows) during regeneration was examined in *E. japonensis* using methyl green-pyronin staining. Soon after fragmentation, neoblasts in the segment(s) at the ends of a fragment divide (K, large arrowheads) and both daughter cells migrate together (L, large arrowheads) leaving their site of previous occupancy vacant (M, small arrowheads). The recovery of neoblasts occurs after regeneration has completed, except for in the anterior-most segment, i.e., the new segment VIII in which neoblasts are absent (N, small arrowheads). g, gut; gz, growth zone; pr, prostomium; py, pygidium; sg, septal (pharyngeal) gland; vnc, ventral nerve cord. Scale bar, 100 µm for (A–B, D, F, H–J), 5 µm for (C, E, G), and 50 µm for (K–N).

Planarian neoblasts are small undifferentiated cells that are richly distributed throughout the body and comprise 20% or more of the somatic cells in an adult worm. They are defined as the only proliferative somatic cells in adult planarians, and differentiate into all cell types including germ cells [6]. In contrast, annelid neoblasts are large cells that are localized at the intersegmental septa along the ventral nerve cord, and number only a few in each segment [5], [18]–[20]. The annelid neoblasts are particularly prominent in oligochaetes that reproduce asexually by fragmentation or fission, and are thought to give rise to mesodermal tissues during regeneration [5], [18]–[20]. However, as the endodermal and ectodermal tissues regenerate via the proliferation of dedifferentiated cells from each layer, the neoblasts are not the only proliferating cells in regenerating annelids. Moreover, many annelid species that lack neoblasts can nonetheless substantially regenerate [19]. An important question that emerges from this therefore is the precise role of the neoblasts in the context of annelid biology. To address this issue in this study, a comparison was made between the regeneration patterns of two enchytraeid oligochaetes (pot worms), *Enchytraeus japonensis* and *Enchytraeus buchholzi*, which possess and lack neoblasts, respectively. Special attention was also paid to regeneration patterns of *E. japonensis* fragments that were amputated within the head region where neoblasts are absent.

**Figure 2:**
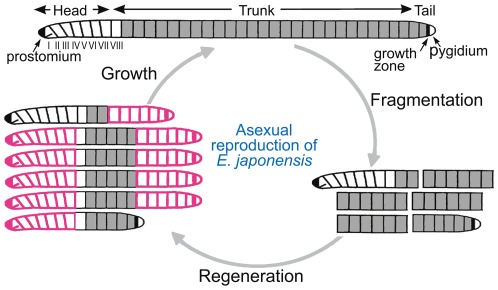
Schematic illustration of the regeneration pattern during asexual reproduction in *E. japonensis*. *E. japonensis* worms harbor neoblasts in all segments except for the anterior-most eight segments, i.e. the seven head-specific segments (segment I–VII) and the first trunk segment (segment VIII). Following spontaneous fragmentation, each fragment regenerates a complete head and/or tail and grows into a normal worm, irrespective of the region of the body from which the fragment was originally derived [4]. Neoblast-bearing segments and regenerated segments are indicated in gray and red, respectively.

It has long been observed that polychaete and oligochaete annelids provide excellent model systems for the study of regeneration [3], [19]. This is principally due to their high regenerative capacity and metameric body structures that enable quantitative measurements of regeneration activity by counting the number of regenerated segments. However, most studies of annelid regeneration have paid no attention to neoblasts, probably because they have dealt with external morphologies or have employed histological analysis of paraffin-sectioned specimens in which neoblasts are often hard to find. In our present study, neoblasts were distinguished unambiguously in whole-mount specimens of *E. japonensis* by staining with RNA affinitive dyes such as methyl green-pyronin (MGP), thionine or propidium Iodide (PI). Using this staining approach, the distribution of neoblasts was closely examined in *E. japonensis* and *E. buchholzi* which are similar in size and morphology, belong to the same genus, and can therefore be analyzed using the same methods. An exact comparison of the regeneration patterns of the two species was thus possible.

Although a considerable number of studies have reported on the regeneration of *E. japonensis* since our proposal of this species as a new model system for regeneration research in 1999 [4], [9]–[15], [17], [21], there has been no report to date on the regeneration of *E. buchholzi*, or of any other enchytraeid that reproduces only sexually. Out of the several hundred enchytraeid species described to date, only eight have been reported to reproduce asexually by fragmentation and subsequent regeneration (see [4] or [10] and literature cited therein). Regeneration has been studied for three enchytraeid species that reproduce asexually by fragmentation and therefore have high regeneration capacities [4], [9]–[15], [18], [22] but not for species that reproduce only sexually. Hence, this is the first study report on the regeneration of a non-fragmenting enchytraeid.

## Results

### Distribution of neoblasts in *E. japonensis* and their absence in *E. buchholzi*

Neoblasts were originally defined as large specialized cells that contribute to mesodermal regeneration in an aquatic oligochaete *Lumbriculus* that reproduces asexually by fragmentation and subsequent regeneration [5]. They are large spindle-shaped cells that locate ventro-laterally on the posterior side of the intersegmental septa along the ventral nerve cord [18], [19] and are characterized by an intensely basophilic cytoplasm and a voluminous nucleus with a prominent nucleolus [19], [20]. In the present study, it was found that neoblasts could be distinguished unambiguously in whole-mount specimens of *E. japonensis* by staining with methyl green-pyronin (MGP). By using this staining method, it became evident for the first time that a pair of neoblasts is located in all segments (Fig. 1A–B) except for the eight anterior-most segments (segments I–VIII), i.e. the seven head-specific segments and the first trunk segment (Fig. 1A). The anterior-most neoblasts are located on the posterior side of the intersegmental septa between segments VIII and IX (Fig. 1A,N). By MGP staining, the neoblasts of *E. japonensis* were revealed to have a large nucleus with a prominent nucleolus and cytoplasm that stains intensely red with pyronin (Fig. 1C). Intense labeling of neoblasts was also found in *E. japonensis* stained with thionine (Fig. 1D,E) and propidium Iodide (PI) (Fig. 1F,G). The intense staining with these three dyes, all of which are known to stain RNA, suggested that the neoblasts are RNA-rich.

In our present study, neoblasts were defined not only by their staining properties but also by their location and morphology. A cell that fulfilled all the following conditions was recognized as neoblast: (1) large and spindle-shaped, (2) located ventro-laterally on the intersegmental septa along the ventral nerve cord, and (3) intensely stained by MGP, PI or thionine. In *E. buchholzi*, none of these staining procedures detected any cells that fulfilled these conditions in any segments in intact individuals (Fig. 1H–J) or in regenerating amputees, indicating that *E. buchholzi* lacks neoblasts in all segments.

**Figure 3:**
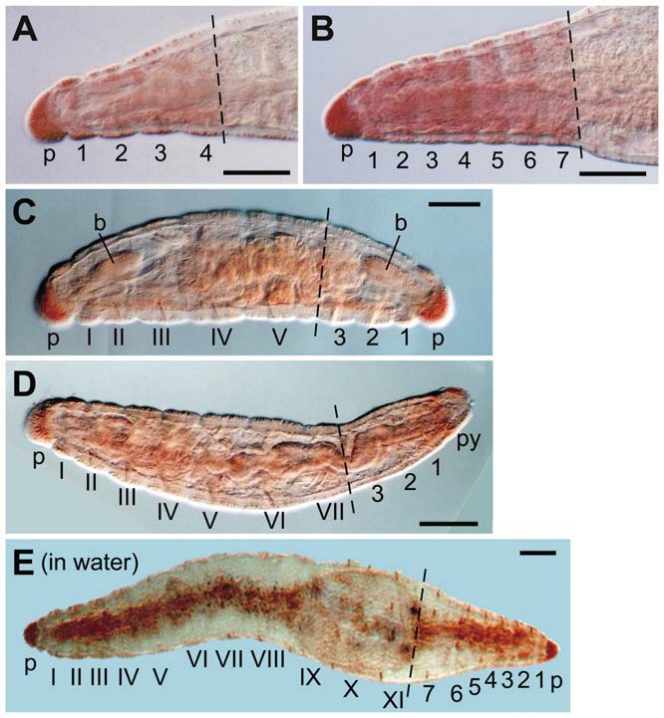
Representative *E. japonensis* regenerates. (A) Example of a head with four segments regenerated after amputation at the 4th–5th segment. (B) A head with seven segments regenerated after amputation in trunk region. (C) A dicephalic monster with biaxial heads formed after amputation at the 6th segment. A head with three segments was regenerated posteriorly in this case. (D) A normal worm regenerated after amputation at the 7th segment. (E) A long dicephalic monster with biaxial heads formed after amputation at the 11th segment and culture in water instead of agar medium. A complete head with seven segments was regenerated posteriorly in this case. Segments of the original fragments are numbered with Roman numerals, and regenerated segments are numbered using Arabic numerals. The broken lines mark the levels of amputation. The anterior is to the left in each image. p, prostomium; py, pygidium. Scale bars, 100 µm.

**Figure 4:**
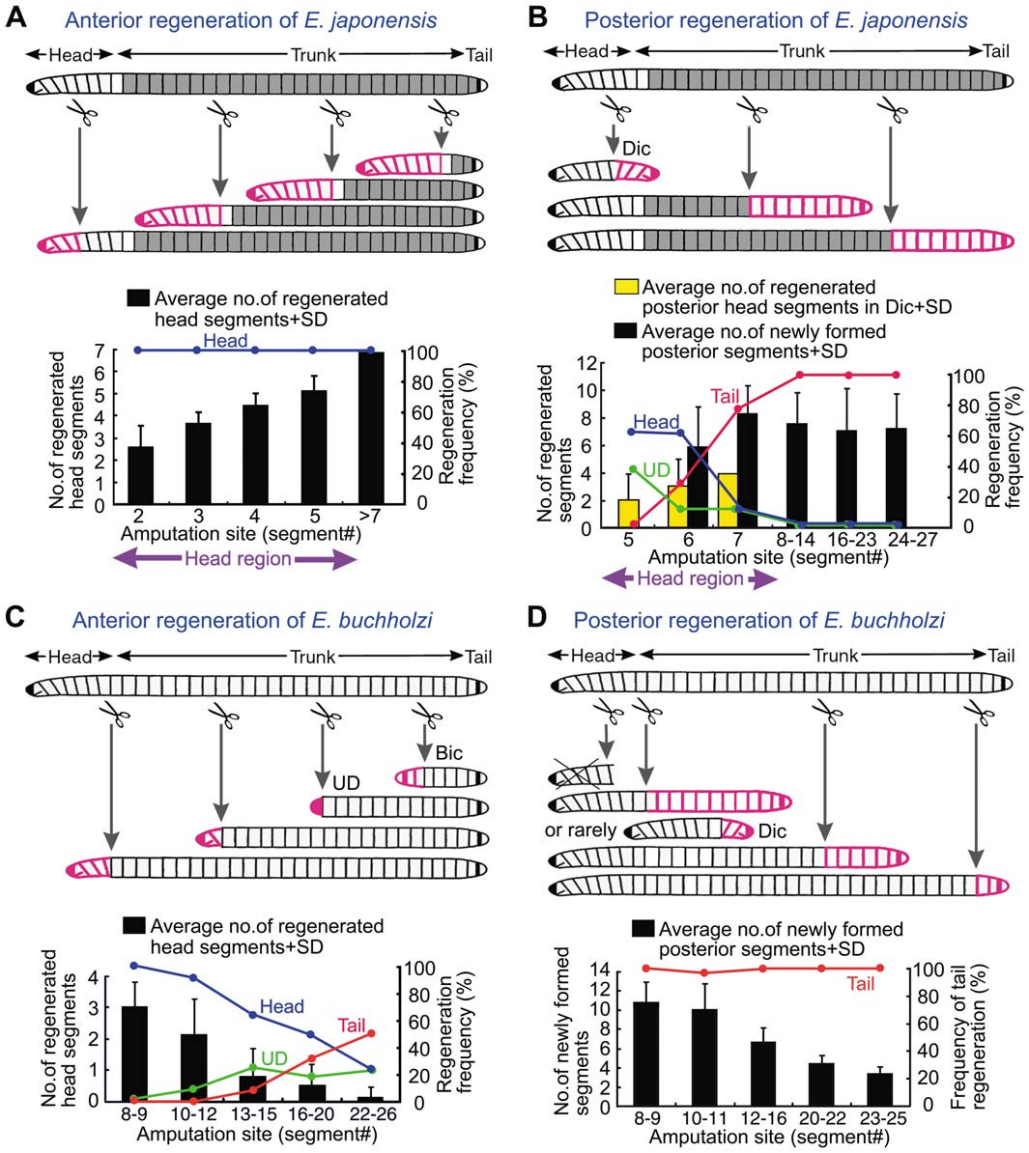
Regeneration patterns of artificially amputated individuals of *E. japonensis* and *E. buchholzi*. The upper illustrations of each panel schematically summarize the results of anterior (A, C) and posterior (B, D) regeneration of *E. japonensis* (A, B) and *E. buchholzi* (C, D) following amputation at various positions along the antero-posterior body axis. Neoblast-bearing segments and regenerated segments are indicated in gray and red, respectively. Fragmentation induced by head removal [4] is omitted from the illustrations of *E. japonensis* to enable an easier comparison with *E. buchholzi*. The lower graphs of each panel show the frequency and degree of regeneration in the anterior (A, C) and posterior (B, D) direction. The bars indicate the mean numbers of regenerated segments with standard deviation (SD), with the numerical axis at the left side. The blue, red and green lines indicate the frequency of regeneration of the head, tail and undeterminable type, respectively, with the numerical axis at the right side. A total of 32, 83, 100 and 109 fragments were examined in (A), (B), (C) and (D), respectively. Bic, bicaudal; Dic, dicephalic; UD, undeterminable.

It has been reported in studies of other neoblast-bearing oligochaetes that shortly after fragmentation or amputation, neoblasts in the segment(s) at both the anterior and posterior ends of the fragments divide and migrate to the amputated sites, where they take part in blastema formation [5], [18], [19], [23]. In this study, by using MGP staining, it was found that at early stages of *E. japonensis* regeneration, both daughter cells of a divided neoblast (Fig. 1K) migrate together (Fig. 1L), leaving behind the site of their previous occupation vacant (Fig. 1M). After regeneration completes, the vacant area is dissolved by the emergence of new neoblasts that are probably derived from the “neoblast-like cells” [15] on intersegmental septa of the segment, but this neoblast recovery did not occur in the anterior-most segment of the stump, i.e., new segment VIII (Fig. 1N). Hence, this segment remains neoblast deficient, as always observed in intact worms (Fig. 1A).

**Table 1:** Comparative summary of the typical regeneration patterns of artificially amputated individuals of *E. japonensis* and *E. buchholzi* cultured in agar medium.

Anterior direction				
	*E. japonensis*		*E. buchholzi*	
Amputation site	Regenerate	Typical result	Regenerate	Typical result
Head region (anterior to 7th segment)	Missing segments	Normal worm	Two to four head segments	Worm with a hypomeric head
Anterior trunk region (8th to ∼14th segment)	Seven head segments (complete head)	Normal worm	One to four head segments	Worm with a hypomeric head
Posterior trunk region (posterior to ∼16th segment)	Seven head segments (complete head)	Normal worm	Tail	Bicaudal monster

Posterior direction				
	*E. japonensis*		*E. buchholzi*	
Amputation site	Regenerate	Typical result	Regenerate	Typical result
Head region (anterior to 7th segment)	Two to five head segments, or occasionally tail (only when amputated in 6th–7th segment)	Dicephalic monster	None	- (incapable of surviving)
Anterior trunk region (8th to ∼14th segment)	Tail and missing segments	Normal worm	Tail and missing segments, or rarely an incomplete head	Normal worm (dicephalic monster in rare cases)
Posterior trunk region (posterior to ∼15th segment)	Tail and missing segments	Normal worm	Tail and missing segments	Normal worm

### Regeneration pattern of *E. japonensis*

Because spontaneous fragmentation never occurs within the head region of *E. japonensis*, all of the fragments produced for asexual reproduction contain neoblasts (Fig. 2). These fragments always regenerate a complete head anteriorly and a complete tail posteriorly, irrespective of the region of the body from which they were originally derived, and regenerated worms grow posteriorly via the addition of new segments in the growth zone (Fig. 2) [4]. Hence, no antero-posterior gradient of regeneration ability exists in the trunk region of *E. japonensis*. It must be noted, however, that *E. japonensis* achieves the regeneration of a complete individual from a body fragment not by a simple restoration of all lost segments, but through a combination of epimorphic recovery of the head and tail and through the morphallactic transformation of old segments into the appropriate segments [4], [13]. This occurs because anterior regeneration in *E. japonensis* is always limited to the seven head-specific segments, no matter how many segments were originally missing (Fig. 2).

**Figure 5:**
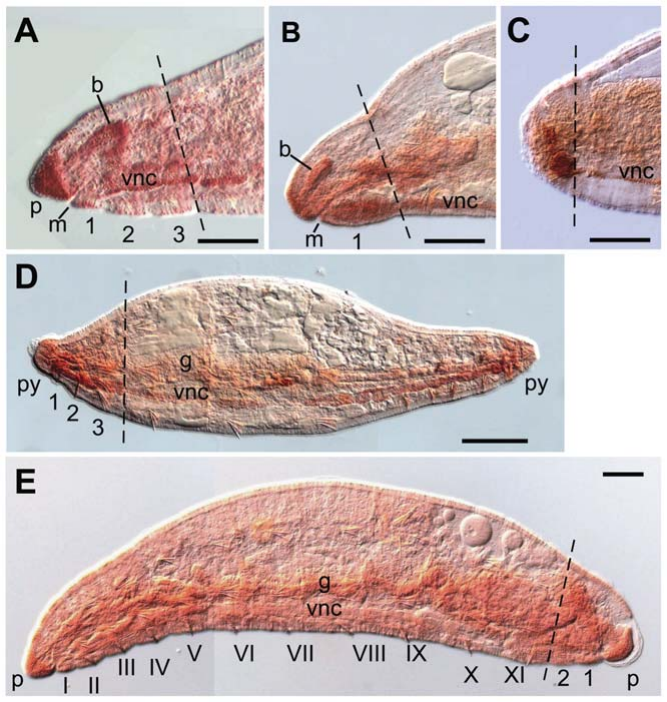
Representative *E. buchholzi* regenerates. (A) Example of a head with three segments regenerated after amputation at the 8th segment. (B) A head with one segment regenerated after amputation at the 12th segment. (C) Undifferentiated blastema of an undeterminable type formed after amputation at around the 20th segment. (D) A bicaudal monster with biaxial tails formed after amputation in the region close to the tail. A tail with three additional segments regenerated anteriorly. (E) A dicephalic monster with biaxial heads formed after amputation at the 11th segments. A head with two segments was regenerated posteriorly in this case. (A–E) Amputees were cultured in 0.6% plain agar for 14 days (A–C), 32 days (E), or 40 days (D), fixed, and then stained with orcein. Segments of the original fragments are numbered with Roman numerals, and regenerated segments are numbered using Arabic numerals. The broken lines mark the levels of amputation. The anterior is to the left and the ventral is down in each image. b, brain; g, gut; m, mouth; p, prostomium; py, pygidium; vnc, ventral nerve cord. Scale bars, 100 µm.

Artificially amputated fragments of *E. japonensis* generally regenerate into normal worms in the same manner as spontaneously divided fragments. However, when amputation was carried out within a head region in which neoblasts are absent, the long posterior amputees anteriorly regenerated the missing segments only (Fig. 3A), instead of the seven head-specific segments (Fig. 3B). The number of head segments that regenerated therefore was dependent on the level of amputation along the body axis in the head region (Fig. 4A, graph). The small anterior amputees, i.e. small head fragments that had been amputated at a site in segments V–VII and thus lacked neoblasts, posteriorly regenerated either a head instead of a tail, resulting in a dicephalic monster (Fig. 3C, Fig. 4B), or a tail, resulting in a normal individual (Fig. 3D, Fig. 4B). This clearly showed that even in the neoblast-bearing *E. japonensis*, both a head and a tail could be regenerated without neoblasts. The number of regenerated head segments however was never larger than five in the dicephalic monsters produced by amputation within the head region (Fig. 4B, yellow bars), suggesting that neoblasts may be indispensable for the regeneration of a complete head.

**Figure 6:**
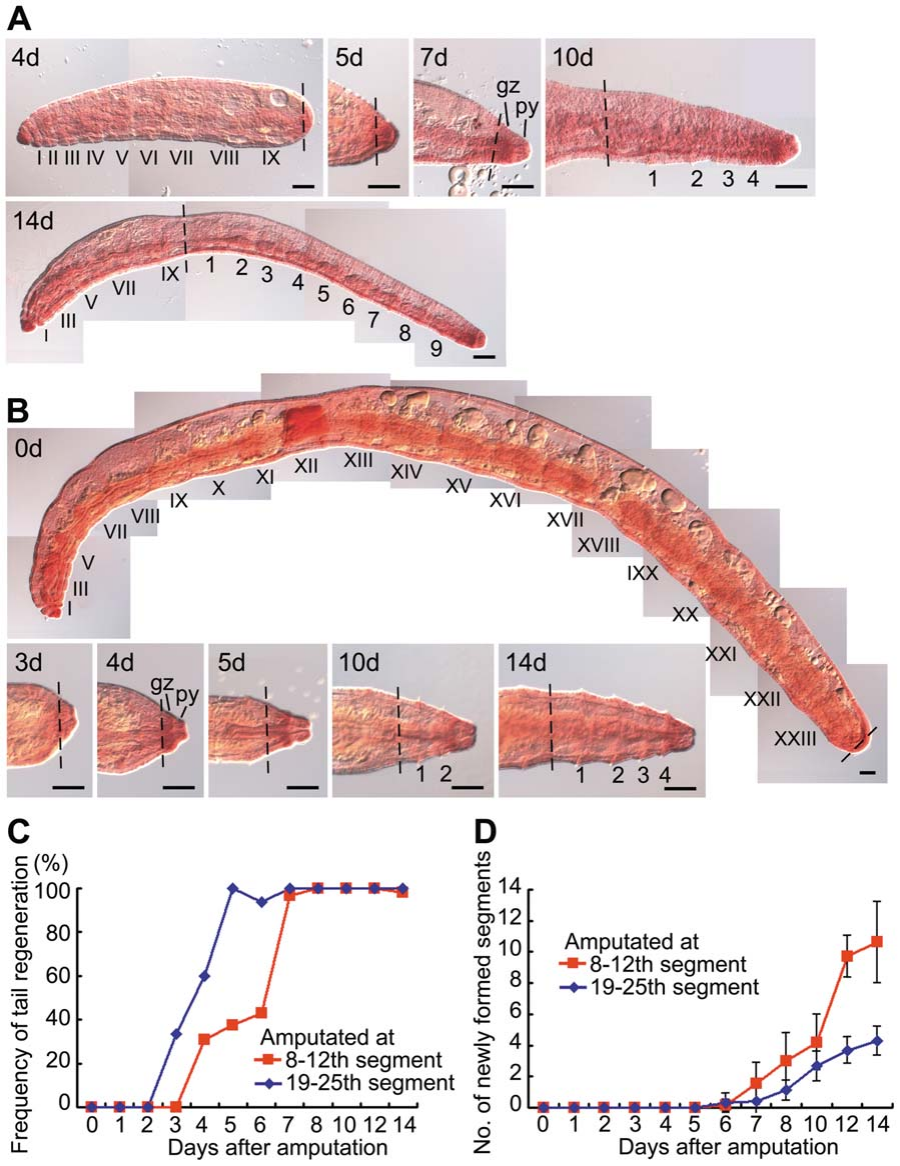
The rapidity of tail regeneration and the extent of the subsequent posterior growth in *E. buchholzi*. (A) Tail regeneration and subsequent posterior growth following amputation at the10th segment in *E. buchholzi*. Regeneration was complete in seven days and nine new segments were formed at 14 days after amputation. (B) Tail regeneration and subsequent posterior growth after amputation at the 24th segment. Regeneration was complete in four days but only four new segments were formed at 14 days after amputation. Segments of the original fragments are numbered with Roman numerals, and regenerated segments are numbered using Arabic numerals. Broken lines mark the levels of amputation. gz, growth zone; py, pygidium. Scale bars, 100 µm. (C, D) Graphs showing that regeneration occurs more rapidly in the posterior region than in the anterior region (C), whilst the extent of posterior growth after tail regeneration is larger in the anterior region than in the posterior region (D). The red and blue lines indicate the frequency of tail regeneration (C) and the mean numbers of newly formed posterior segments with standard deviation (D) after amputation at the 8th–12th segment and the 19th–25th segment, respectively. A total of 222 and 152 fragments were examined in (C) and (D), respectively.

**Figure 7:**
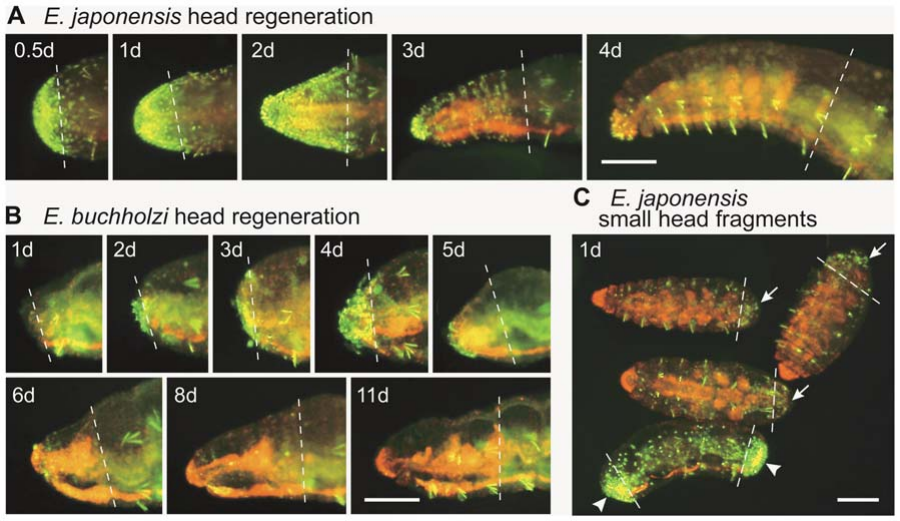
Cell proliferation activity during head regeneration in *E. japonensis* and *E. buchholzi*. (A) Spontaneous fragments from the trunk region of *E. japonensis*. (B) Posterior fragments of *E. buchholzi* that were amputated at the 5th–9th segment. (C) Small head fragments of *E. japonensis* that were amputated at the 6th–7th segments (upper three specimens, with arrows indicating weakly-labeled blastemas) and a spontaneous fragment from the trunk region (lower specimen, with arrowheads indicating strongly-labeled blastemas). Fragments were incubated at 23°C, labeled with BrdU for 18 hours, fixed and immunostained for BrdU (yellow dots) and counterstained with propidium Iodide (orange). Chaetae show intense yellow autofluorescence signals. The days after amputation (including BrdU labeling time) are indicated. Broken lines mark the levels of amputation. Scale bars, 100 µm.

When amputation was carried out in the trunk region in which neoblasts are present, the anterior and posterior amputees always regenerated a tail posteriorly and a head anteriorly, resulting in a complete individual (Fig. 4A,B). However, when amputees were cultured in water instead of agar medium, 40% (41 out of 102 examined) of the anterior amputees posteriorly regenerated a head resulting in a long dicephalic monster (Fig. 3E). This occurred probably because “corrective autotomy”, which is thought to be important for tail regeneration to occur after artificial amputation, is inhibited in water [21]. In these long dicephalic monsters, a complete head with seven segments was regenerated posteriorly (Fig. 3E), possibly because neoblasts were present in posterior segments of these amputees.

These results suggested that in *E. japonensis*, each of the head segments that lacks neoblasts has a different positional identity according to its position along the antero-posterior body axis (Fig. 4A,B) and that there is an antero-posterior gradient of regeneration ability in the head region. In contrast, the segments in the trunk region, which contain neoblasts, adopt the same positional identity (of segment VIII) after fragmentation or amputation with respect to their regeneration ability, so that fragments from any body region can regenerate a complete individual (Fig. 2, Fig. 4A,B, Table 1). Moreover, the results of the experiments dealing with dicephalic monsters suggested that neoblasts may be essential for regeneration of a complete head with seven segments.

### Regeneration pattern of *E. buchholzi* – anterior direction

In *E. buchholzi*, head regeneration occurred only when amputation was carried out within or near to the head region (Fig. 4C, Fig. 5A–C). Moreover, the numbers of regenerated head segments decreased with the distance of the amputation site from the original head (Fig. 4C). The range of the numbers of regenerated head segments was 0–4, 0–2, and 0–1 when amputation was carried out in the 4th–14th, 15th–19th, and 21st–30th segments, respectively. In the hypomeric heads, the prostomium and brain were also found to be reduced in size (Fig. 5B).

When amputations were undertaken in the region close to the tail, the small posterior amputees anteriorly regenerated a tail instead of a head, resulting in a bicaudal monster with biaxial tails (Fig. 4C, Fig. 5D). Such a phenomenon never occurs in *E. japonensis* [4], [21] but is known to occur in some other annelids [24], [25].

These results suggested that the anterior segments within or near to the head in *E. buchholzi* have a higher potential for head regeneration, whilst the posterior segments near to the tail have a higher capacity for tail regeneration than the segments of other regions (Fig. 4C, Table 1).

### Regeneration pattern of *E. buchholzi* – posterior direction

When amputation was carried out within the head region (in the 3rd–7th segments), the small anterior amputees died within seven days without regeneration (none of the 48 examined specimens survived). When amputation was performed immediately posterior to the head segments (in the 8th–11th segments), the amputees generally regenerated a tail, resulting in a normal individual, but on rare occasions (two out of 38 specimens examined), a head instead of a tail was regenerated, resulting in a dicephalic monster with biaxial heads (Fig. 4D, Fig. 5E). When amputations were carried out in the trunk region, the anterior fragments always regenerated a tail irrespective of the amputation position (Fig. 4D). There was still an antero-posterior gradient of regeneration ability however as tail regeneration occurred more rapidly in the posterior region than in the anterior region, whilst the extent of posterior growth after tail regeneration decreased as the amputation site neared the original tail i.e. if more segments were removed by amputation, more new segments were formed in the growing areas after tail regeneration (Fig. 4D, Fig. 6).

These results suggested that each segment in *E. buchholzi* has a different positional identity throughout the body with respect to its regenerative ability and that this accords with its position along the antero-posterior body axis. The differing regenerative potentials in different body regions applied to regeneration in both the anterior and posterior direction.

### Comparison of cell proliferation activity during the anterior regeneration of *E. japonensis* and *E. buchholzi*

It was found in our analyses that anterior regeneration blastemas of *E. buchholzi* were smaller than those of *E. japonensis* and that regeneration proceeds much more slowly in *E. buchholzi* than in *E. japonensis* (Fig. 7A,B). To examine whether these phenomena are due to lower cell proliferation activity in the blastemas of *E. buchholzi* compared with those in *E. japonensis*, BrdU labeling experiments were performed. In *E. japonensis*, active cell proliferation began soon after fragmentation and continued at high levels until blastemal segmentation occurred at three days after fragmentation (Fig. 7A). In contrast, in *E. buchholzi*, cell proliferation activity was very low throughout the regeneration processes except at four days after amputation when the regeneration blastema was formed (Fig. 7B). To assess whether the low proliferation activity observed in *E. buchholzi* was correlated with the absence of neoblasts, BrdU labeling was monitored in small head fragments of *E. japonensis* that had been artificially amputated within the head region and thus lacked neoblasts. It was found that cell proliferation activity was very low in the regeneration blastemas of these small head amputees (Fig. 7C, arrows) in comparison with those of trunk region amputees in which neoblasts were present (Fig. 7C, arrowheads). These results suggest that neoblasts may contribute to the active proliferations of blastemal cells and to the rapid formation of blastemas with the potential to regenerate a complete head in *E. japonensis*.

**Table 2:** Comparison of the biological and species characteristics of *E. japonensis* and *E. buchholzi*.

	*E. japonensis*	*E. buchholzi*
Size of full-grown worms in laboratory culture	10 mm (50–70 segments) long×0.2 mm thick	10 mm (30–40 segments) long×0.3–0.4 mm thick
Distribution	Reported only from Japan [33]	Widely distributed around the world [29], [30]
Mode of reproduction	Asexually by fragmentation and sexually under certain conditions [4]	Only sexually
Self-fertilization	Incapable (M. Myohara, unpublished data)	Capable [31]
Autotomy	Frequent fragmentations for asexual reproduction	Capable for detoxification [35]
Neoblasts	Present in all segments except for the eight anterior-most segments	Absent in all segments

## Discussion

As posterior regeneration has been documented in numerous annelid species including those that have been shown to lack neoblasts [19], [24], [26], it seems obvious that unlike the situation in planarians, neoblasts are dispensable for regeneration in annelids. The analyses in our present study further address the precise functional roles of annelid neoblasts with the aim of providing greater clarity around the differences between the properties of these cells and those of the planarian neoblasts. Self-renewal and pluripotency, the fundamental properties that define a stem cell, have been experimentally determined in planarian neoblasts [2], but not yet in annelid neoblasts. Nevertheless, because they have the same name, annelid neoblasts have sometimes erroneously been regarded as pluripotent somatic stem cells although no experimental evidence has been presented indicating that they have any stem-cell characteristics other than an unspecialized cytological appearance. Indeed, in situ hybridization studies have in fact now shown that the *piwi* gene, which encodes a key regulator of stem cell self-renewal in various organisms including planarians [8], [27], is not expressed in *E. japonensis* neoblasts [12], [15]. Moreover, alkaline phosphatase activity, which is commonly used as a marker for pluripotent stem cells in vertebrates, has also not been detected in *E. japonensis* neoblasts [10]. These findings suggest that *E. japonensis* neoblasts may not be pluripotent stem cells. Actually, based on their examination of regeneration of a naid oligochaete, Bilello and Potswald have commented that the neoblasts may simply represent a peritoneal stem cell population that is restricted to regenerating peritoneally derived tissues and should not be considered pluripotent reserve cells [23]. It thus seems more likely that if there are multipotent stem cells in *E. japonensis*, they would be located in the growth zone from which both germ cells and neoblasts may originate, as shown previously in the polychaete annelid *Platynereis dumerilii* [28]. Although pluripotent/totipotent adult somatic stem cells are rare, tissue-restricted adult stem cells (such as the neural stem cells and the intestinal stem cells) are common in many animals. It therefore is probable that other tissue-restricted adult stem cells besides neoblasts are present in *E. japonensis* and in *E. buchholzi*. Needless to say, much more studies must be carried out to characterize the neoblasts and other stem cells in annelids.

In our present study, comparative analyses of regeneration patterns were carried out between two closely related oligochaete annelids with special regard to the distribution of neoblasts. The results show that the neoblast-bearing species, *E. japonensis*, which can reproduce asexually by fragmentation, has the ability to regenerate a complete head at any body level where neoblasts are present, whilst the neoblast-lacking species, *E. buchholzi*, which cannot reproduce asexually, never regenerates a complete head. Moreover, an antero-posterior gradient of regeneration ability is discernible in *E. buchholzi*, but not in *E. japonensis* (apart from the head region). These results suggest that, with respect to regenerative ability, the neoblast-bearing segments of *E. japonensis* adopt the same positional identity (of the 8th segment) after fragmentation or amputation, whereas each neoblast-lacking segment in *E. japonensis* (i.e. the eight anterior-most segments) and in *E. buchholzi* (all segments) have a different positional identity that accords with its position along the body axis. Although little is currently known about the cellular and molecular basis of the annelid morphogenetic gradient, it is speculated that the nervous system, which is organized for transmission in an antero-posterior direction, plays an important role in this phenomenon [24].

The results of our present study also reveal that cell proliferation in the regenerative blastemas begins later and is less active in *E. buchholzi* than in *E. japonensis*. This results in the formation of smaller blastemas with a limited regenerative potential in *E. buchholzi*. Cell proliferation was also found to be less active in the blastemas of *E. japonensis* small head amputees, which lack neoblasts and never regenerate a complete head. This suggests that neoblasts may contribute to the rapid formation of blastemas with the potential to regenerate a complete head in *E. japonensis*. This enables any fragment with neoblasts to regenerate a complete individual. In contrast, in neoblast-lacking segments, cell dedifferentiation seems to occur over a period of time before cell proliferation begins leading to the slow formation of blastemas.

Our results clearly show the correlation between the presence of neoblasts and the absence of morphogenetic gradient. Correlation is also clear between the presence of neoblasts and the active cell proliferation in the regeneration blastemas with high regenerative potential. However, it remains to be accounted for how neoblasts are actually concerned with these phenomena.

Our current data suggest that *E. buchholzi*, as well as *E. japonensis*, can be an effective model system for future regeneration studies. Both *E. japonensis* and *E. buchholzi* are small terrestrial enchytraeids that are very easy to culture in the laboratory. Their small size and thin, transparent cuticles make it easier to perform whole-mount observations of morphology and gene expression patterning (e.g. [9], [14], present study). In addition, as these two species belong to the same genus, they are phylogenetically quite closely related, similar in size and morphology, and can thus be analyzed using the same methods. However, they have several distinct characteristics (Table 2) that have implications for their usage as an experimental model system. The wide global distribution of *E. buchholzi* [29], [30] makes it readily accessible, particularly as the international transportation of live animals faces considerable restrictions. Moreover, the ability of *E. buchholzi* to self-fertilize [31], together with its short generation time (two weeks at 24–25°C), allows for the rapid establishment of pure lines in only a few months [32] (M. Myohara, unpublished data). After repeating selfing six times, the probability of homozygosis at any one locus is >98.4% in each of these lines [32]. The availability of such lines will be advantageous in future genetic and molecular studies of regeneration in annelids. Previously we have isolated 165 genes that were upregulated during regeneration of *E. japonensis* by cDNA subtraction cloning [9]. Comparisons of regeneration-upregulated genes between *E. japonensis* and *E. buchholzi* may lead to the identification of genes that are related to function of annelid neoblasts.

In conclusion, our present results argue for the first time that the presence of neoblasts in annelids correlates with the absence of an antero-posterior gradient of regeneration ability along the body axis, and suggests that the annelid neoblasts are more essential for efficient asexual reproduction than for the regeneration of missing body parts. In addition, *Enchytraeus buchholzi* is proposed as a new model system for future regeneration studies.

## Materials and Methods

### Worms

*Enchytraeus japonensis* [33] and *Enchytraeus buchholzi* [29] worms were provided by Y. Nakamura and have been maintained in our laboratory since 1995 and 1998, respectively. These worms were reared in 1.1% (w/v) plain agar medium in 150×15 mm disposable Petri dishes at 23–24°C, and fed with rolled oats. Under these conditions, *E. japonensis* grows continuously to about 10 mm in length, consisting of 50–70 segments, and reproduces asexually by fragmentation approximately every two weeks [4]. Under these same conditions, *E. buchholzi* also grows to about 10 mm in length, consisting of 30–40 segments, but reproduces only sexually. Embryogenesis is completed in both species at 5–6 days after oviposition [10], and juveniles hatched from the cocoon become mature worms and begin to lay eggs at 14 days after oviposition. In both species also, the head comprises seven heteronomous segments that are equipped with specific organs such as the mouth, brain, pharynx, or septal (pharyngeal) glands, and the tail is the pygidium with an anteriorly adjacent growth zone [34].

### Whole-Mount Staining

For methyl green-pyronin (MGP) staining, specimens were fixed in 70% ethanol overnight at room temperature and stained in MGP solution (HT70-1; Sigma Diagnostics, St Louis, MO, USA) for 40–60 minutes. For thionine staining, specimens were fixed and stained briefly in 0.5% thionine (Nakarai Chemicals, Kyoto, Japan) in 45% acetic acid for one minute, and then in 0.5% thionine in 70% ethanol for 3–5 minutes, washed in 70% ethanol and then in water. For propidium Iodide (PI) staining, specimens were fixed in 4% paraformaldehyde in phosphate buffered saline (PBS) for 30 minutes at room temperature, washed in PBS for 10 minutes four times, permeabilized with 0.2% Triton X-100 (Sigma Chemical Co, St. Louis, MO, USA) in PBS for 30 minutes, washed in PBS, stained with 2 µg/ml PI (P-4170, Sigma) in PBS for 20 minutes at room temperature, and again washed in PBS. For orcein staining, specimens were fixed in freshly prepared AGE fixative (acetic acid: glycerol: ethanol = 4∶1∶2) for 15–20 minutes, stained in 4% orcein (Merck, Darmstadt, Germany) in AG (acetic acid: glycerol = 4∶1) for 20 minutes, and washed with AGE. Stained specimens were whole-mounted in 50% glycerol in water (for MGP, thionine, and orcein) or in 50% glycerol in PBS (for PI), and examined under a microscope equipped with a differential interference contrast (DIC) (Axiophot 2, Carl Zeiss, Germany). The images were captured using a digital camera system (AxioCam, Carl Zeiss).

### Investigation of Artificially Amputated Fragments

Artificial amputations of *E. japonensis* and *E. buchholzi* were carried out using needle-sharp tweezers (T-4412, Sigma) and fine-tip dissection scissors (Napox R-12, Natsume Seisakusho Co., Tokyo, Japan), respectively. The amputees were cultured in 0.6% (w/v) plain agar medium in 35×10 mm disposable Petri dishes at 23–24°C for 4–5 days in the case of *E. japonensis* or for 14–40 days for *E. buchholzi*. The worms were then fixed and stained with orcein and examined under a DIC-microscope as described above.

### BrdU Labeling and Detection

Fragments of *E. japonensis* and *E. buchholzi* at five hours to 14 days after amputation were incubated in distilled water containing 20 mM bromodeoxyuridine (BrdU; B-5002, Sigma) for 18 hours at 23°C. To detect BrdU uptake immunohistochemically, specimens were fixed in 4% paraformaldehyde in PBS for one hour at room temperature, washed in PBS, treated with 2N HCl for 30 minutes, neutralized with 0.1 M Na_2_HPO_4_ (pH 8.5) for 15 minutes twice, washed in PBS, blocked and permeabilized by incubation in PBS containing 1% bovine serum albumin (BSA, B-4287, Sigma) and 0.2% Triton X-100 for 30 minutes. The samples were then labeled with an anti-BrdU monoclonal antibody (MAB3510, Millipore, Jaffrey, NH, USA) diluted 1∶20 in 0.2% Triton X-100 in PBS for 2–4 days at 4°C, then washed in PBS. BrdU antigen was visualized by incubation with FITC-labeled anti-mouse IgG (F8521, Sigma) diluted 1∶50 in 0.2% Triton X-100 in PBS for four hours at room temperature. The samples were then washed in PBS, counterstained in 2 µg/ml PI (P-4170, Sigma) in PBS for 10 minutes at room temperature, washed again in PBS, and whole-mounted in 50% glycerol in PBS, examined under a fluorescence dissection microscope (MZ 16F, Leica Microsystems GmbH, Wetzlar, Germany) and photographed using a digital camera system (VB-7000, Keyence, Osaka, Japan).
